# Danon disease presenting with transient stroke-like weakness in a young woman: a case report

**DOI:** 10.3389/fcvm.2026.1735648

**Published:** 2026-02-24

**Authors:** Jiao Wang, Xiaokai Zhou, Lixia Zhou, Yaoyao Ruan, Jinhua Wang, Qizhi Jin

**Affiliations:** 1 Department of Cardiology, The Quzhou Affiliated Hospital of Wenzhou Medical University (People’s Hospital of Quzhou), Quzhou, Zhejiang, China; 2 Department of Neurology, The Quzhou Affiliated Hospital of Wenzhou Medical University (People’s Hospital of Quzhou), Quzhou, Zhejiang, China

**Keywords:** cardioembolism, cryptogenic stroke, Danon disease, dilated cardiomyopathy, heart failure, LAMP2

## Abstract

**Background:**

Danon disease is a rare X-linked dominant lysosomal storage disorder caused by lysosome-associated membrane protein 2 (LAMP2) deficiency. Female carriers demonstrate highly variable penetrance, and neurological manifestations are under-recognized, which delays diagnosis and targeted management.

**Case presentation:**

A 27-year-old woman presented with sudden-onset left-sided weakness that resolved within three weeks. Initial magnetic resonance imaging demonstrated a right basal ganglia infarction with distal right middle cerebral artery narrowing, and she received dual antiplatelet therapy and statins. When she was referred for etiologic evaluation, physical and neurological examinations were unremarkable, yet N-terminal pro-B-type natriuretic peptide and cardiac troponin I levels were elevated. Transthoracic echocardiography revealed left ventricular dilation with global hypokinesia (ejection fraction 30%). Right heart contrast echocardiography excluded patent foramen ovale, whereas 24 h Holter monitoring captured frequent atrial and ventricular ectopy with short ventricular tachycardia runs. Cardiac magnetic resonance showed markedly reduced systolic function (left ventricular ejection fraction 21%) and mid-wall late gadolinium enhancement, while myocardial perfusion imaging confirmed global hypoperfusion. Whole-exome sequencing identified a heterozygous LAMP2 frameshift variant (c.1079_1083delGAAAG; p.Gly360Valfs*11), which was validated by Sanger sequencing. Cascade testing revealed a hemizygous carrier son and wild-type parents. She was treated with contemporary heart failure therapy, oral dabigatran for presumed cardioembolic stroke, and listed for heart transplantation.

**Conclusion:**

This case underscores that Danon disease in women may initially mimic cryptogenic stroke. Multimodality cardiac imaging combined with genetic analysis is crucial for recognizing atypical presentations and guiding anticipatory heart failure management.

## Introduction

Danon disease (OMIM: 300257) is an X-linked dominant lysosomal storage disorder resulting from LAMP2 deficiency. Male patients commonly experience childhood-onset hypertrophic cardiomyopathy, skeletal myopathy, and cognitive impairment, whereas female heterozygotes exhibit delayed and heterogeneous manifestations (1, 2). Neurological symptoms are seldom emphasized in women, leading to misdiagnosis and treatment delays. We describe a young woman whose initial presentation was a transient stroke-like episode, subsequently attributed to Danon disease. The case highlights how comprehensive cardiac assessment and genetics refine diagnosis, risk stratification, and transplant timing in atypical female carriers.

## Case presentation

A 27-year-old right-handed woman presented to a local hospital after awakening with sudden left-sided clumsiness, leg dragging, dizziness, and somnolence. Brain magnetic resonance imaging (MRI) revealed diffusion restriction in the right basal ganglia, and magnetic resonance angiography showed distal right middle cerebral artery narrowing. The working diagnosis was a large-artery atherosclerotic stroke, and she was started on aspirin 100 mg daily, clopidogrel 75 mg daily, and atorvastatin 20 mg nightly. Neurological deficits resolved within three weeks.

Seeking clarification for the stroke etiology, she was admitted to our neurology service on March 18, 2019. She reported two months of exertional dyspnea, fatigue, and recurrent upper-respiratory infections but denied chest pain, syncope, or prior neuromuscular symptoms. She had no history of hypertension, diabetes, dyslipidemia, or substance use. Family history was notable only for maternal hypertension. She had delivered a son by cesarean section at age 21. Physical examination revealed a body temperature of 36.6°C, heart rate 88 beats/min, blood pressure 108/78 mmHg, and respiratory rate 18 breaths/min. Cardiac auscultation revealed a soft holosystolic murmur at the apex; neurological examination, including strength and coordination, was normal. She measured 151 cm tall and weighed 55 kg, corresponding to a body mass index of 24.1 kg/m^2^ and estimated body surface areas of 1.593 m^2^ by the Chinese adult formula and 1.472 m² by the Stevenson equation.

Laboratory testing showed creatine kinase 299.1 U/L (reference 105–245), urine protein 1+, and elevated N-terminal pro-B-type natriuretic peptide 1,677.9 pg/mL (reference < 100) with mildly increased cardiac troponin I 0.063 ug/L (reference < 0.026). Liver function, renal function, coagulation profile, autoimmune antibodies, and infectious serologies were unremarkable.

**Figure 1:**
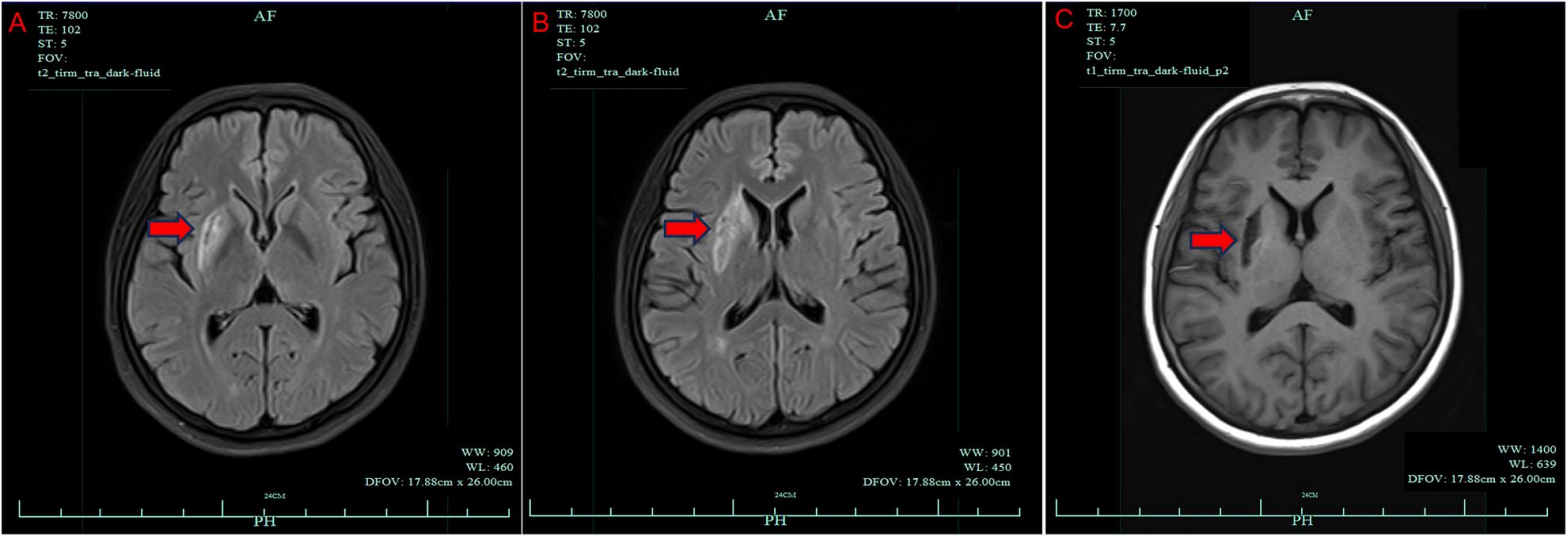
Follow-up brain MRI on admission. **(A)** Diffusion-weighted sequence illustrating persistent hyperintensity in the right basal ganglia. **(B)** FLAIR image depicting the subacute infarct extent. **(C)** T1-weighted image indicating low signal within the infarct core.

**Figure 2:**
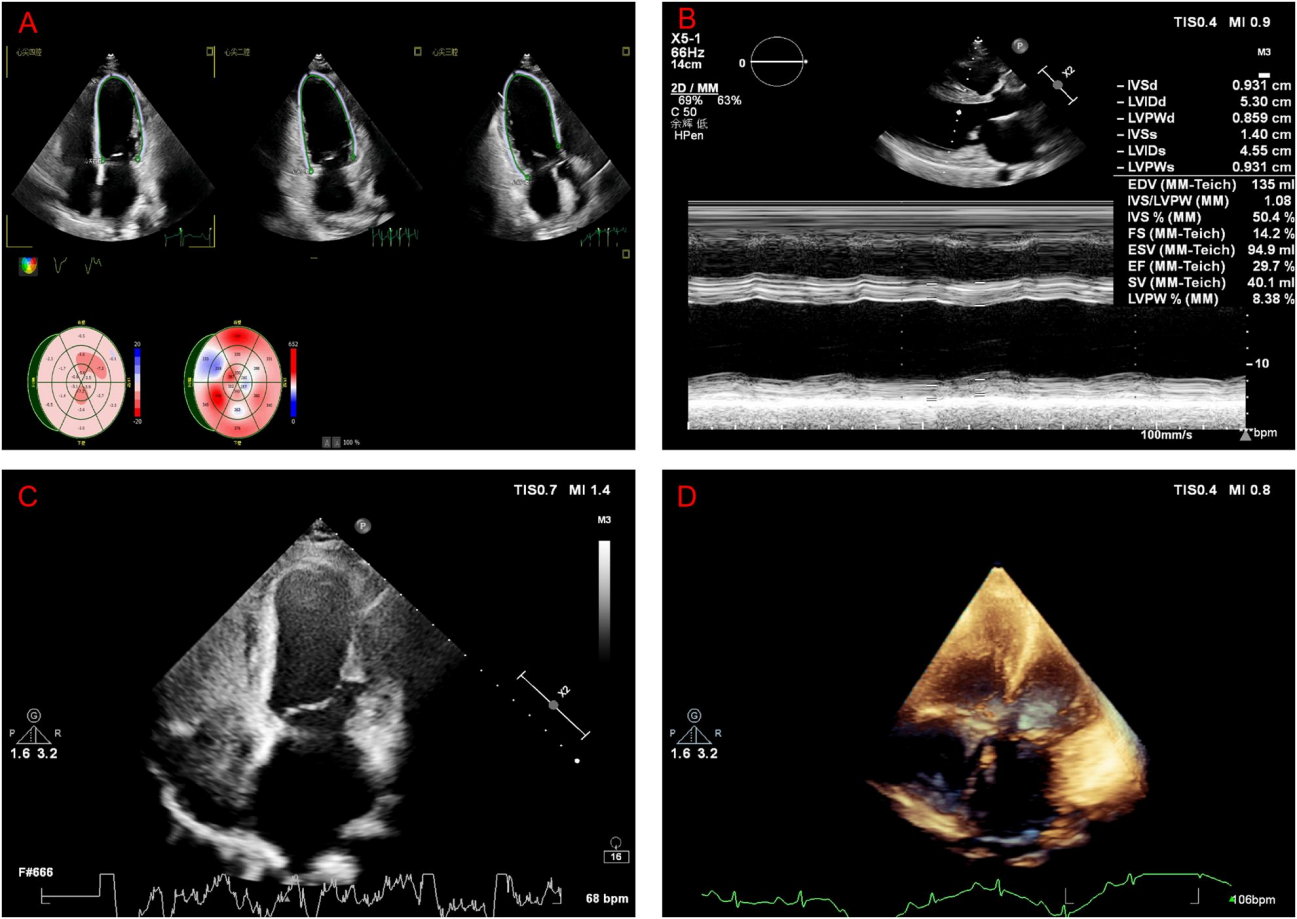
Echocardiography and contrast findings. **(A,B)** Transthoracic echocardiography showing a dilated left ventricle with diffuse hypokinesia (LVIDd 63 mm, LVEF 30%) in orthogonal views. **(C,D)** Right heart contrast echocardiography demonstrating no right-to-left shunt at rest or during the Valsalva maneuver.

Repeat brain MRI confirmed a subacute right basal ganglia infarction without hemorrhagic transformation (Figures 1A–C). Transthoracic echocardiography demonstrated left atrial enlargement (43 mm), left ventricular end-diastolic diameter 63 mm, end-systolic diameter 53 mm, interventricular septum 10.3 mm, posterior wall 11.6 mm, and a reduced left ventricular ejection fraction (LVEF) of 30% with diffuse hypokinesia (Figures 2A,B). Mild mitral and tricuspid regurgitation and elevated pulmonary artery pressure were present. Right heart contrast echocardiography under resting and Valsalva conditions revealed no right-to-left shunt, excluding patent foramen ovale or atrial septal defects (Figures 2C,D). Three-dimensional echocardiography corroborated global dysfunction with a volumetric LVEF of 30%, left ventricular end-diastolic and end-systolic diameters of 5.3 and 4.6 cm, respectively, severely blunted global longitudinal strain (−3.0%), and an estimated right ventricular systolic pressure of 49 mmHg without pericardial effusion. Indexed chamber assessment yielded a three-dimensional left ventricular end-diastolic volume of 120 mL (75 mL/m^2^) with cavity dimensions of 6.1 × 4.5 cm, underscoring proportionate dilation after normalization to body surface area.

Twenty-four-hour Holter monitoring recorded sinus rhythm with an average ventricular rate of 83 beats/min (range 60–132 beats/min), 804 premature atrial contractions with occasional bigeminy or trigeminy, 1,584 premature ventricular contractions including 22 couplets, and two brief runs of non-sustained ventricular tachycardia. High-peaked P waves with intraventricular conduction delay were observed.

**Figure 3:**
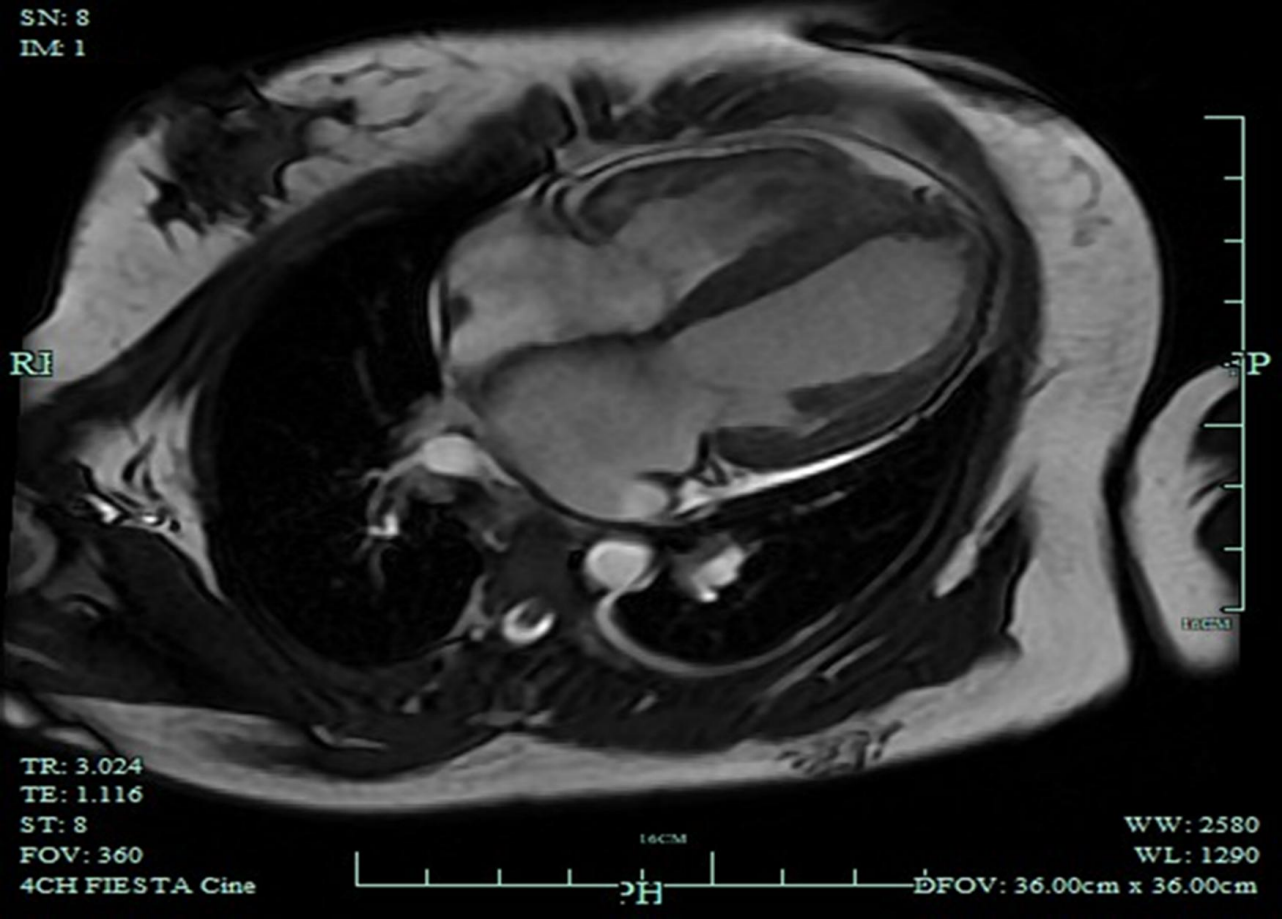
Multimodality cardiac imaging. Late gadolinium enhancement cardiac MRI displaying patchy mid-wall fibrosis with severely reduced systolic function (LVEF 21%).

Cardiology consultation on March 20, 2019 recommended evaluation for cardiomyopathy. Cardiac magnetic resonance imaging (March 22, 2019) revealed left ventricular dilation (end-diastolic diameter 66.8 × 59.0 mm), global systolic dysfunction (LVEF 21%), preserved right ventricular size with reduced function, and patchy mid-wall late gadolinium enhancement of the septal, anterior, lateral, and inferior walls (Figure 3). Myocardial perfusion single-photon emission computed tomography demonstrated globally decreased tracer uptake with prominent apical defects. These findings supported advanced dilated cardiomyopathy with diffuse fibrosis and impaired viability. Coronary angiography was deferred because of presumed non-ischemic cardiomyopathy and normal electrocardiographic ischemia markers. Dedicated coronary CT angiography was not pursued because duplex examinations documented normal carotid and intracranial arteries and the mediastinal window of contrast-enhanced chest CT showed smooth epicardial coronaries without calcification or caliber change, making obstructive coronary atherosclerosis highly unlikely in this young woman.

**Figure 4:**
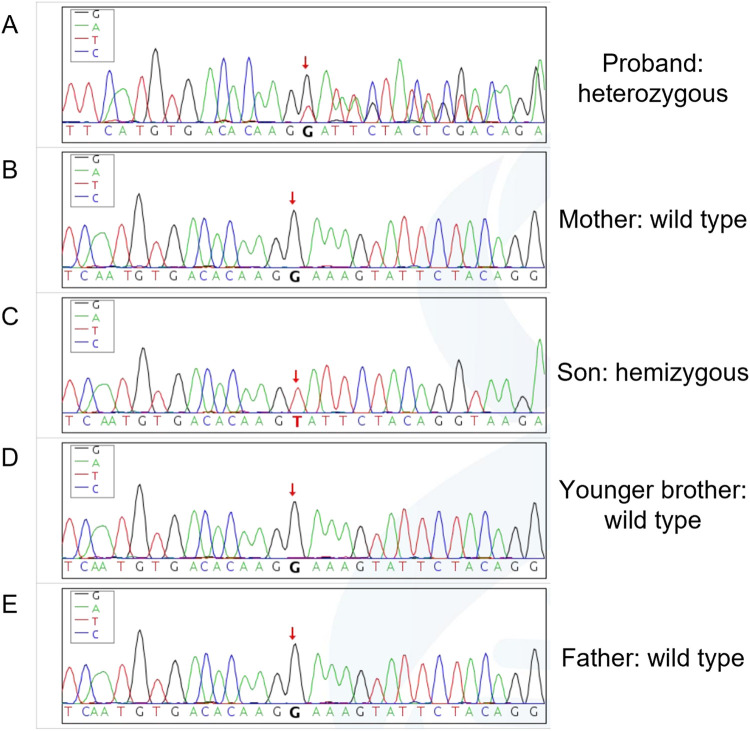
Family-based Sanger confirmation. **(A)** Proband chromatogram showing the heterozygous LAMP2 c.1079_1083delGAAAG (p.Gly360Valfs*11) deletion. **(B)** Son chromatogram demonstrating the hemizygous deletion. **(C)** Maternal chromatogram showing wild-type sequence across the locus. **(D)** Paternal chromatogram showing wild-type sequence. **(E)** Younger brother chromatogram confirming wild-type status.

Given the unexplained dilated cardiomyopathy and embolic stroke in a young woman, genetic testing was pursued. Whole-exome sequencing achieved a mean coverage of 132x with 99.4% of targeted bases at >=20x and identified a heterozygous frameshift variant in LAMP2 (NM_002294.3: c.1079_1083delGAAAG), predicted to generate a premature termination codon (p.Gly360Valfs*11). Sanger sequencing confirmed the heterozygous LAMP2 c.1079_1083delGAAAG (p.Gly360Valfs*11) deletion in the proband (Figure 4A). Cascade testing demonstrated the hemizygous deletion in her son (Figure 4B), whereas her mother, father, and younger brother retained wild-type alleles (Figures 4C–E). Additional rare variants detected in BBS1, DCHS1, VWF, RYR2, and CYP4V2 were interpreted as variants of uncertain significance without phenotypic correlation. Although the commercial laboratory initially categorized the LAMP2 variant as uncertain significance, application of American College of Medical Genetics and Genomics criteria (PVS1, PM2, PM6) alongside the family segregation data supported reclassification to likely pathogenic.

Multidisciplinary consensus favored cardioembolic stroke secondary to severe Danon cardiomyopathy. The patient initiated comprehensive guideline-directed medical therapy, including sacubitril/valsartan, metoprolol succinate, spironolactone, dapagliflozin, torsemide, and vericiguat. Long-term oral anticoagulation with dabigatran 110 mg twice daily replaced dual antiplatelet therapy. She received counseling regarding heart transplantation and was placed on the waiting list. At the latest follow-up (18 months), she reported NYHA class III symptoms despite optimized therapy and ongoing transplant evaluation.

## Discussion

Danon disease arises from loss of LAMP2, a lysosomal membrane protein critical for autophagic flux and intracellular homeostasis (3). Female heterozygotes frequently experience later onset and milder phenotypes than males because of random X-chromosome inactivation, yet life-threatening cardiomyopathy often develops in adulthood (4). This case illustrates that stroke-like episodes may precede overt heart failure symptoms in women, consequently delaying recognition of Danon disease. Unlike the more classically described concentric or asymmetric hypertrophic cardiomyopathy, our proband manifested a predominantly dilated cavity with relatively preserved septal thickness, which may reflect variant-specific expression of the LAMP2 c.1079_1083delGAAAG frameshift and mirrors the comparable severity across genders noted by Pasqualucci et al. (5).

The patient's transient neurological deficit and imaging findings initially suggested large-artery atherosclerotic stroke. However, absence of vascular risk factors, normal vascular imaging on follow-up, and presence of severe left ventricular dilation with diffuse fibrosis favored a cardioembolic mechanism. Late gadolinium enhancement in Danon cardiomyopathy typically localizes to the subepicardial or mid-wall regions and corresponds to replacement fibrosis that promotes arrhythmia and thromboembolism (6). Frequent ventricular ectopy and impaired systolic function further increased embolic risk in this patient.

The frameshift variant c.1079_1083delGAAAG truncates the luminal domain of LAMP2 and has not been previously reported in ClinVar or HGMD. Frameshift and nonsense variants constitute the majority of pathogenic alleles and usually correlate with severe cardiac involvement (7). Although the testing laboratory issued an initial classification of uncertain significance, the loss-of-function mechanism, absence from population datasets, and segregation in the pedigree satisfied ACMG PVS1, PM2, and PM6 criteria, supporting likely pathogenicity. Detection of the same variant in the patient's son underscores the need for early surveillance in asymptomatic carriers. Conversely, the absence of the variant in both parents suggests a *de novo* event or parental germline mosaicism.

From a management perspective, early identification of Danon disease enables timely initiation of guideline-directed medical therapy, arrhythmia surveillance, and transplant planning. Although enzyme replacement is unavailable, investigational gene therapies targeting LAMP2B show promise (8). Implantable cardioverter-defibrillators are often considered in male patients, yet risk stratification in females remains debated. The patient's non-sustained ventricular tachycardia and extensive fibrosis warrant consideration of device therapy during transplant evaluation. Her combination of a left ventricular ejection fraction of 21%, extensive mid-wall late gadolinium enhancement, and documented non-sustained ventricular tachycardia are established sudden cardiac death markers in Danon cardiomyopathy (1), so we initiated intensified Holter and telemetry surveillance and are evaluating prophylactic implantable cardioverter-defibrillator placement while she awaits transplantation.

This case adds to the limited literature describing neurologic presentations of Danon disease in women. Clinicians evaluating cryptogenic stroke in young adults should inquire about subtle cardiomyopathy symptoms and obtain echocardiography when indicated. Multimodality imaging combined with next-generation sequencing can unmask rare genetic cardiomyopathies and refine therapeutic strategies.

## Conclusion

Danon disease should be considered in young women who present with unexplained stroke-like episodes accompanied by cardiomyopathy. Comprehensive cardiac imaging, arrhythmia monitoring, and genetic testing are pivotal for accurate diagnosis, prognostication, and transplant referral.

## Patient perspective

The patient expressed relief at obtaining a unifying diagnosis and was willing to pursue heart transplantation. She is concerned about her son's carrier status and is receiving genetic counseling for long-term surveillance.

## Data Availability

The authors acknowledge that the data presented in this study must be deposited and made publicly available in an acceptable repository, prior to publication. Frontiers cannot accept a manuscript that does not adhere to our open data policies.
